# Colorectal cancer (CRC) as a multifactorial disease and its causal correlations with multiple signaling pathways

**DOI:** 10.1042/BSR20200265

**Published:** 2020-03-20

**Authors:** Mao-lin Wan, Yu Wang, Zhi Zeng, Bo Deng, Bi-sheng Zhu, Ting Cao, Yu-kun Li, Jiao Xiao, Qi Han, Qing Wu

**Affiliations:** 1 Department of Hepatobiliary and Pancreatic Surgery, Xianning Central Hospital, the First Affiliated Hospital of Hubei University of Science and Technology, Xianning, 437000, P.R. China; 2 Department of Laboratory Medicine, The Second Affiliated Hospital of University of South China, Hengyang, 421001, P.R. China; 3 Department of Pathology, Xianning Central Hospital, the First Affiliated Hospital of Hubei University of Science and Technology, Xianning, 437000, P.R. China; 4 Department of Oncology, Xianning Central Hospital, the First Affiliated Hospital of Hubei University of Science and Technology, Xianning, 437000, P.R. China; 5 Department of Digestive Medical, The Affiliated Nanhua Hospital, University of South China, Hengyang, 421002, P.R. China; 6 Key Laboratory of Tumor Cellular and Molecular Pathology, College of Hunan Province, Cancer Research Institute, University of South China, Hengyang, Hunan, 421001, P.R. China; 7 Department of Endocrinology, The Affiliated Nanhua Hospital, University of South China, Hengyang, 421002, P.R. China

**Keywords:** CRC, interaction, metastasis, proliferation, signaling pathway

## Abstract

Colorectal cancer (CRC) is the third most common malignancy and one of the leading causes of cancer-related death among men worldwide. CRC is a multifactor digestive pathology, which is a huge problem faced not only by clinicians but also by researchers. Importantly, a unique feature of CRC is the dysregulation of molecular signaling pathways. To date, a series of reviews have indicated that different signaling pathways are disordered and have potential as therapeutic targets in CRC. Nevertheless, an overview of the function and interaction of multiple signaling pathways in CRC is needed. Therefore, we summarized the pathways, biological functions and important interactions involved in CRC. First, we investigated the involvement of signaling pathways, including Wnt, PI3K/Akt, Hedgehog, ErbB, RHOA, Notch, BMP, Hippo, AMPK, NF-κB, MAPK and JNK. Subsequently, we discussed the biological function of these pathways in pathophysiological aspects of CRC, such as proliferation, apoptosis and metastasis. Finally, we summarized important interactions among these pathways in CRC. We believe that the interaction of these pathways could provide new strategies for the treatment of CRC.

## Introduction

Colorectal cancer (CRC) is the third most common malignancy worldwide and is one of the leading causes of cancer-related death [1]. The high mortality rate is reflective of several factors, including the lack of apparent symptoms in the early stages of CRC and the absence of cancer prevention strategies in developing countries, which causes enormous economic and psychological burden for people worldwide [1,2]. Although surgical resection achieves good clinical efficacy for localized cases, the median 5-year overall survival rate is only approximately 66.4%, which is attributed to the vast majority of CRC cases being medically diagnosed in a late, incurable stage, and the effects of traditional cancer chemotherapy are limited [2].

CRC is a multifactorial digestive pathology and is an enormous problem facing not only clinicians but also scientific researchers [3]. Many investigators believe that inflammation, *Helicobacter pylori* infection, dynamics of gut microbiota, hormonal disorders, gene mutation, epigenetic modification, immune dysfunction and metabolic disturbance are the main and classical risk factors for CRC [4–9]. Several molecular mechanisms, including advanced glycation end product (AGE) [10], aberrant glycosylation [11], abnormal telomerase activity [12], unfolded protein response (UPR) [13], angiogenesis [14], reactive oxygen species (ROS) production [15], epithelial–mesenchymal transition (EMT) [16], cell apoptosis, proliferation, survival, migration, invasion, self-renewal, differentiation and dedifferentiation reprogramming, are altered to survive host defense or therapeutic insults. However, the dysregulation of these molecular mechanisms may not explain CRC origins and development, suggesting that various genetic and epigenetic events occur at the gene level [17,18].

The function and interaction of molecular pathways have a significant role in multiple cancer types. Previous studies have indicated that CRC progression is mediated by the dysregulation of many signaling pathways, including Wnt [19], PI3K/Akt [20,21], Hedgehog [22], ErbB [23], RHOA [24], Notch [25], BMP [26], Hippo [27], AMPK [28], NF-κB [29], MAPK [3] and JNK [30]. Moreover, the interaction of these pathways is precise and complicated. In addition, a growing body of research shows that genetic perturbation or epigenetic dysregulation can promote the development of CRC or that the cancer itself can provoke genetic perturbation or epigenetic dysregulation [18]. Vdovikova et al. found that bacteria can target host cell epigenetics to promote carcinogenesis in HCT8 cells [31]. Daeun et al. suggested that β-carotene could inhibit DNMT3A and global DNA methylation levels to decelerate CRC progression [32]. Wu and his colleagues summarized that epigenetics play an important role in CRC progression [33].

In the current review, we summarize recent progress in studying these important potential molecular mechanisms and highlight their impact on CRC in order to reveal an attractive therapeutic strategy for CRC in the near future.

## Multiple carcinogenic and anticarcinogenic intracellular pathways in CRC

**Figure 1:**
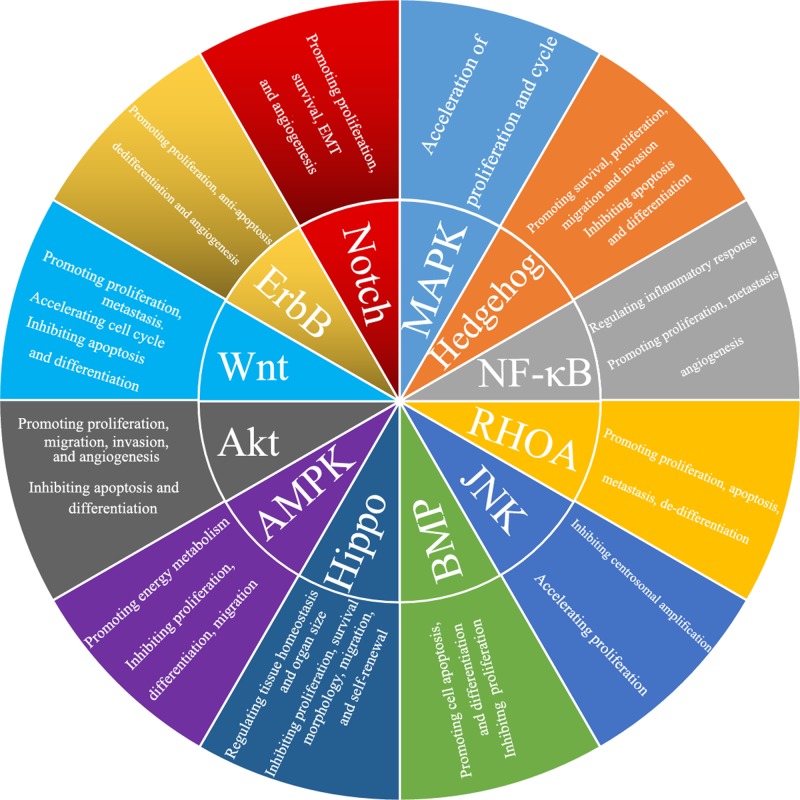
The function of these signaling pathways in CRC Wnt, PI3K/Akt, Hedgehog, ErbB, Notch, NF-κB and MAPK can promote the carcinogenesis of CRC. BMP, AMPK and Hippo can inhibit the development and progression of CRC. However, RHOA and JNK may play dual roles in CRC.

To explore the molecular pathogenesis of CRC, we summarize recent progress in CRC (Figure 1). The intracellular signaling pathways contributing to carcinogenesis have been elaborated, and the driver genes have been considered promising targets for tumor therapy. Moreover, increasing research on molecular disorders of CRC provides valuable insight into the carcinogenesis of CRC, which may be explained by several molecular mechanisms playing multiple roles at different stages or in different situations during cancer development.

### Up-regulated Wnt signaling pathway promotes CRC progression

Wnt signaling can be divided into two types: canonical and noncanonical. In the canonical Wnt pathway, Wnt is engaged with its receptors LRP-5/6 and Frizzled, which also activates Disheveled (DVL), recruiting the complex (Axin, GSK-3β, CK1, APC) to the receptor, which then impedes cytosolic GSK-3β rendering it incapable of phosphorylating β-catenin, resulting in the accumulation of unphosphorylated β-catenin in the cytosol [34–36]. Unphosphorylated β-catenin then translocate to the cell nucleus, where it interacts with T cell-specific factor (TCF)/lymphoid enhancer-binding factor (LEF) and coactivators, including Pygopus (Pygo) and Bcl-9, to turn on Wnt target genes such as c-Myc, cyclin D1 and Cdkn1a [34–36].

One of the noncanonical Wnt signaling pathways, the planar cell polarity pathway (PCP), is primitively activated through the interaction between Wnt and Frizzled receptors, along with coreceptors DAAM1/2, to control the activity of small GTPases, including RhoA, which play a role in regulating cytoskeletal remodeling [37]. Rho-associated kinase (ROCK) and myosin are motivated by Rho GTPase to rearrange the cytoskeleton and actin. Activated Rac GTPase and Rac activate the Jun N-terminal kinase (JNK) pathway [24]. Another noncanonical Wnt signaling pathway is the Wnt/Ca^2+^ pathway, which is activated by Wnt5a. Guanine nucleotide binding protein (G-protein) is cleaved into protein β/γ subunits and G-protein alpha-t2 via frizzled FZD2, resulting in Ca^2+^ influx into cytoplasm to promote differentiation. Moreover, Ca^2+^ can promote calmodulin and CaMKII to promote the phosphorylation of T-cell factor and lymphoid enhancer factor, which can repress the canonical Wnt pathway [38–41].

The level of YAP, a key gene in the Hippo pathway, is increased by the β-catenin/TCF4 complexes that bind to the first intron of the YAP gene DNA enhancer element in CRC cells [27]. The first report of the cross-talk between Wnt and Notch was in fruit flies, which indicated that Wnt could be controlled by Notch [27]. The Notch pathway activated by the APC mutant is necessary for the initiation and progression of colonic polyps and subsequent tumors in mice [42]. Kwon and his colleagues [43] found that β-catenin could physically interact with the membrane-bound cytoplasmic tails of Notch. In a previous study, it was identified that the Ras pathway can be up-regulated by enhancing Ras stability in CRC by mutations in APC (WNT pathway). The author found that Ras stability was regulated by Wnt activity, regulating Ras recruitment to the proteasome [44].

### The PI3K/Akt signaling pathway is enhanced and accelerates carcinogenesis in CRC

The PI3K/Akt signaling pathway is obviously up-regulated in CRC, and inhibition of this pathway may provide a potential therapeutic approach that may induce curable CRC [45,46]. Glucose metabolism is closely related to this signaling pathway in CRC. PI3K, a lipid kinase, promotes the phosphorylation of phosphatidylinositol-4,5-bisphosphate (PIP2) into phosphatidylinositol-3,4,5-trisphosphate (PIP3), in turn facilitating Akt phosphorylation to induce an intracellular signaling cascade [47]. In addition, AKT subtypes have convergent and divergent functions during tumorigenesis. PIP3 is degraded and its signal is terminated by phosphoinositol phosphatases, including PTEN, PIPP (INPP5J) and INPP4B. The PI3K pathway contributes to the growth and transformation of cancer cells and is mediated by proteins called phosphoinositol phosphatases, which are usually dysregulated in human cancers [48].

This pathway can also phosphorylate NF-κB to facilitate cell survival, which phosphorylates and degrades IκB (an inhibitor of NF-κB). Therefore, liberated NF-κB translocates to the nucleus to enhance cell survival and thus induce carcinogenesis. NF-κB not only enhances survival but also induces angiogenesis [49], which also plays a significant role in CRC progression induced by PI3K/Akt/eNOS [50]. In addition, PI3K/Akt can promote the phosphorylation of MDM2, an oncogenic protein, which can promote the ubiquitination of p53. p53 is a significant tumor suppressor gene that accelerates cellular apoptosis in response to DNA damage from ionizing radiation or stress stimulation [51]. In CRC, the Fas/Fas-ligand system is an important downstream gene-expression cascade for the PI3K/Akt signaling pathway, which induces cellular apoptosis. Fas and Fas-ligand, members of the tumor necrosis factor receptor family, can recruit the Fas-associated death domain to induce cell apoptosis by activating the caspase cascade [52,53]. In addition, PI3K/Akt can also enhance cell survival by phosphorylating Bad, a proapoptotic protein, to degrade it, which can increase the level and activity of the antiapoptotic proteins Bcl-xl and Bcl2 [54]. p27Kip1 (p27), a significant regulator of cyclin-dependent kinase 2, induces the G1/S transition, which can cooperate with p130Rb2 (p130) to inhibit the cellular cycle at the G1/S transition [55,56]. In addition, PI3K/Akt can obviously inhibit the expression of p27 and p130 to accelerate the cell cycle by inhibiting forkhead protein [57]. Furthermore, PI3K/Akt can deactivate glycogen synthase kinase 3 (GSK3) to increase the levels of cyclin D1 and myc, accelerating the cell cycle to upregulate proliferation [58].

Furthermore, the PI3K/Akt pathway can inhibit the Hippo pathway by promoting the phosphorylation of YAP to accelerate colon cancer cell proliferation [59]. In addition, the PI3K/Akt pathway can activate mTOR to promote protein synthesis, resulting in cell metabolism, growth and proliferation [46,60]. Other work has implicated interactions between PI3K/Akt signaling and BMP in colon cancers [61]. Taken together, these results suggest that this pathway contributes to CRC development.

### The ErbB signaling pathway plays an oncologic role in CRC

The receptor tyrosine kinase superfamily is mainly distributed on the surface of CRC and breast cancer cells and plays a significant role in nearly every aspect of cell biology. The ErbB receptor family has four subtypes, including ErbB1 (EGFR), ErbB2 (HER2), ErbB3 (HER3) and ErbB4 (HER4) [62].

In a recent study, EGFR was overexpressed in 60–80% of colon cancers [62,63]. In addition, a standardized IHC test was used for the clinical trial analysis to separate CRC patients who expressed EGFR in at least 1% of the cancer cells [64,65]. EGFR triggers a molecular cascade that results in activation of the MAPK and PI3K signaling pathways, which can promote the proliferation, apoptosis inhibition, dedifferentiation and angiogenesis of CRC cells [66].

Park and his colleagues found that 47.4% (65/137) of patients with CRC exhibited overexpression of HER2, which was closely associated with poor prognosis [67]. Activation of HER2 is predominantly present in CRC cells, mediating differentiation, proliferation and apoptosis [68]. Furthermore, constant amplification of HER2 can mediate chemoresistance by activating ERK1/2 signaling [69]. HER2 is the preferred partner of other ErbBs, and the formation of dimers or complexes with HER3 and HER4 is involved in the initiation, development and progression of cancer [70].

HER3, as an impaired kinase in the receptor tyrosine kinase superfamily, is phosphorylated only by dimerization with another ErbB receptor [70,71]. This most often happens to HER2, which is the most carcinogenic member of the family [72]. Baiocchi showed a 69.7% HER3 response rate in patients with CRC, resulting in lymph vascular invasion [73]. In addition, HER3 is expressed in many tumors that express HER2 [74]. Therefore, the HER2/HER3 axis may play a significant role in the aberrant growth of CRC cells [75].

HER4, activated by heparin-binding EGF-like growth factor, neuregulins and betacellulin, can activate the PI3-kinase and Shc pathways to promote cell proliferation and metastasis but inhibit differentiation [76]. Daekee Lee found that inhibition of HER3 leads to increased caspase-3-mediated apoptosis in cancers, and absence of HER3 or HER4 leads to CRC cell apoptosis, which may be caused by the HER3-HER4 heterodimer-dependent AKT pathway. Furthermore, they observed that activation of PI3K/AKT is the primary mechanism promoting CRC growth in an EGFR–HER3 heterodimer-dependent manner [17].

In mouse CRC cells that have both WNT and Ras activating mutations, Christopher observed that ectopic HER4 expression enhances unanchored growth *in vitro* and the establishment of xenografts *in vivo*. Moreover, HER4 knockout disrupted the WNT-induced growth of CRC cells. These results suggest that high levels of HER4 coexist with activated WNT signaling to promote carcinogenesis of mouse and human colon cells. Furthermore, ectopic expression of HER4 is also correlated with EGFR pathway activation in human colorectal cancer [19]. Interestingly, EGFR can also activate the Ras–Raf–MEK–ERK signaling pathway in colon cancer, which accelerates the progression and development of colon cancer [3].

### The dual function of the RHOA signaling pathway in CRC development

Many investigators believe that the RHOA signaling pathway, in its carcinogenic role, has a complex interaction with many cancers via proliferation, apoptosis, metastasis, dedifferentiation and polarization [77–79], although a recent study suggests that it has totally different function in CRC than it does in other cancers [80]. In previous studies, many investigators showed that activation of RHOA can promote the proliferation and growth of CRC [77,81]. Nevertheless, Paulo Rodrigues and his colleagues found that decreases in the levels of RHOA have a strong correlation with poor patient prognosis [80,82], and inactivation of RHOA induced obviously faster proliferation, metastasis, dedifferentiation and polarization *in vivo* and *in vitro* [80]. These data suggest that there are two different theories about the activation state of RHOA: one is that activation of RHOA directly increases the proliferation and growth of cancer cells [77,81]. The other, in opposition, is that inactivation of RHOA depends on Rho-associated protein kinase (ROCK) and diaphanus-related formin 1 (DIAPH1) to up-regulate canonical Wnt signaling to maintain constant proliferation and growth [80]. In general, the suggestion that inactivation of RHOA can promote carcinogenesis is presumably because canonical Wnt signaling is a master regulator of carcinogenesis in CRC, and this pathway can induce constant proliferation and growth [83,84].

### The Notch signaling pathway plays an oncogenous role in the development and progression of CRC

The Notch pathway is activated in multiple cancer types as a result of multiple genetic changes, such as point mutations, gene amplification, chromosomal translocations and other epigenetic modifications [85]. It interacts with four receptors, Notch 1/2/3/4, to regulate cell proliferation, differentiation, apoptosis and stem cell maintenance [86]. Different studies in CRC patients have hinted that the Notch pathway is implicated in the development of CRC, and a series of mechanisms have been proposed to demonstrate this regulation [87,88]. First, the Notch intracellular domain (NICD) is cleaved from the receptor by an γ-secretase complex [87], and the cleaved NICD translocates from the cytoplasm to the nucleus [88]. Second, NICD interacts with recombining binding protein suppressor of hairless (RBPJ), resulting in the activation of oncogenes, such as hairy enhancer of split (HES) family proteins, CDKN1A (also known as p21), HES-related proteins (HEY), Notch-regulated ankyrin repeat protein, cyclin D1/3, c-myc and HER2 [89,90]. Many investigators have demonstrated that the Notch pathway is up-regulated in patients with CRC, which ultimately induces cancer cell proliferation, survival, EMT and angiogenesis [87,91,92]. Notch ligand expression is increased in several solid tumors, including CRC and pancreatic cancer. In addition, there appears to be a close interaction between the Notch and Ras signaling pathways, as Ras activating mutations have been shown to activate Notch signaling, which is required for Ras-mediated transformation [93].

### Bone morphogenetic protein (BMP)/SMAD pathways as important tumor suppressor pathways in CRC

It is generally accepted that BMP4, a member of the transforming growth factor-β (TGF-β) superfamily, plays a significant role during embryonic development that regulates cell apoptosis, proliferation and differentiation [26]. Regarding the function of BMP4 in cancers, BMP4 has been indicated to mediate the differentiation of cancer stem cells and inhibit the cancer growth of CRC [94].

BMP, a secreted glycoprotein, activates and binds to BMPR1A (ALK3), BMPR1B (ALK6) and BMPRII receptors by activating Drosophila (SMAD) 1/5/8 and phosphorylated Mothers against decapentaplegic [95]. Subsequently, activated p-SMAD 1/5/8 translocates from the cytoplasm to the nucleus by forming a complex with SMAD4, and ultimately, it regulates transcription and expression of target genes such as inhibitor of DNA binding (ID) [96] and CCL15 [97]. Importantly, the BMP pathway is down-regulated in the majority of colon cancer cases [98].

A recent report indicated that β-catenin can be inhibited by BMP via SMAD4 to down-regulate the expression of *c-myc* and *Axin2* in microdissected small intestinal adenomas of tamoxifen-treated mice [98]. Another study sufficiently suggests that BMP and WNT appear to be interconnected via the PI3k/Akt pathway [99,100].

### The Hedgehog signaling (HH) pathway plays a tumorigenic role in CRC

HH signaling was first detected in *Drosophila melanogaster* and was demonstrated to play a significant role in mediating proliferation, establishing cellular fate and regulating embryonic development [101]. The HH family is a group of proteins implicated in many cellular functions: cell survival, proliferation, apoptosis, differentiation, migration and invasion [102]. Hedgehog (SHH, IHH or DHH) binds to the receptor PTCH1, which is a direct target of Hedgehog signaling [103]. This interaction can relieve PTCH1-mediated repression of SMO, which can subsequently activate a downstream signaling pathway, leading to the translocation of GLI1 and GLI2 (GLI family zinc-finger transcription factors) from the cytoplasm to the nucleus [104], where they regulate the transcription of several target genes, including platelet-derived growth factor (*PDGF*) [105] and *SNAIL* [106].

Hh signaling is involved in some types of solid tumors, especially basal cell skin cancer and medulloblastoma. Notably, the HH signaling pathway plays a key role in the progression and repair of colon epithelial cells, and a previous study indicated that it was activated in CRC. Various studies have reported up-regulation of HH ligand SHH, HH receptor PTCH1, and hh-associated transmembrane receptor Smoh in hyperplastic polyps, adenomas, and adenocarcinomas of the colon [93]. Exogenous SHH can promote the growth of colon cells in primary mouse models, suggesting that shh-triggered signals may promote the development of CRC. The study also showed that the expression level of Shh mRNA in CRC cells was significantly higher than that in normal colon cells [107].

### The Hippo signaling pathway involved in the anticarcinogenesis of CRC

The Hippo signaling pathway is a significant signaling pathway in the regulation of stem cell proliferation, morphology, survival, migration, self-renewal, migration, tissue homeostasis and organ size [108–110]. Recently, Hippo signaling has been considered one of the most significant signaling pathways in tumorigenesis, as it inhibits the development of tumors though multiple components of this pathway, including fat storage-inducing transmembrane protein, large tumor suppressor kinase (LATS), macrophage stimulating (MST), taffazin (TAZ), Yes-associated protein 1 (YAP1) and transcriptional enhancer associated domain (TEAD) [111]. In brief, MST increased by the transmembrane protein of FAT upregulates LATS to phosphorylate YAP, which prevents the translocation of YAP from the cytoplasm to the nucleus. Ultimately, it attenuates the ability of the YAP/TAZ complex to interact with TEAD [112–114], resulting in the inactivation of oncogenes such as β-catenin, k-ras, and Akt/mTOR in colon tumor initiation and progression [115–117].

In CRC, the Hippo signaling pathway is suppressed, and the expression of YAP is increased, which induces the migration, invasion, proliferation and EMT of colon cancer cells [16]. The interaction between Ck1δ/ε and Dvl is impeded by TAZ, which blocks Dvl phosphorylation mediated by Wnt3a and finally represses Wnt/β-catenin signaling. Furthermore, dysregulation of MST and LATS also blocks the membrane translocation of TAZ to block the pathophysiological effect of Wnt3a [118]. In addition, YAP1/KLF5 can bind to the promoter of Achaete scute-like 2 (Ascl2), which is a Wnt signaling target, and transcriptionally enhance its expression, resulting in increased self-renewability of CRC progenitor cells [119]. In colon-derived cell lines, inhibition of YAP obviously decreased the effect of Notch and Wnt signaling to reduce proliferation and survival ability. In Mst1/2-deficient cells, the expression of Hes1 and Hey1 (key regulatory genes in the Notch pathway) was largely enhanced [120]. Moreover, YAP overexpression induced Notch ectopic activation to inhibit cell differentiation [121].

### The function and mechanism of AMPK, a key energy metabolism pathway, in CRC

AMPK, a serine/threonine kinase, is characterized by sensitivity to changes in the ratio of AMP/ATP; AMPK acts as a cellular energy sensor, regulating various cell progression processes such as CRC cell survival, proliferation, differentiation, migration and metabolism [122,123].

At the molecular level, the major effects of AMPK are predominantly exerted through the regulation of oxidative phosphorylation and the interaction of other signaling pathways. In particular, activation of AMPK can activate p53, which induces autophagy and apoptosis and decelerates the cell cycle. Phosphorylated AMPK inhibits the phosphorylation of mTOR to inhibit cell proliferation and protein synthesis by mediating tuberous sclerosis 1 and 2 (TSC1/2) [20,21], inactivating Rag GTPases [124] and up-regulating the expression of REDD1 [125]. Furthermore, activation of AMPK negatively regulates receptor tyrosine kinase pathways, such as the ErbB2 and EGFR pathways, further targeting the downstream protein factors mTOR and ERK [23]. Inactivated AMPK can also inhibit insulin receptor substrate-1 (IRS1), which is an activating factor for the IGF1/insulin signaling axis and subsequent PI3K/Akt/mTOR signaling, which can inhibit neoplastic activity [126]. AMPK activation by metformin recruits anti-angiogenic and anti-inflammatory factors, such as interleukin-1β (IL-1β), tumor necrosis factor α (TNFα), IL-6, nuclear factor κ light-chain-enhancer of activated B-cells (NF-κB) and hypoxia inducible factor-1α (HIF-1α), diminishing the effect of vascular endothelial growth factor (VEGF) [127,128]. AMPK, as an upstream regulator of the Hippo pathway, can also promote the phosphorylation of YAP to inhibit its effects on colon cancer cells, including proliferation, apoptosis inhibition, glucose uptake and glycolysis [28].

On the other hand, AMPK displays obvious metabolic effects that are associated with malignancy, such as the development of a lipogenic phenotype. Phosphorylation of AMPK can facilitate the phosphorylation of acetyl-coenzyme A carboxylase-1 (ACC-1) to decrease the levels of fatty acid synthase (FASN), sterol-regulatory element-binding protein (SREBP-1c) and stearoyl coenzyme A desaturase-1 (SCD-1) [127,129], thereby impeding lipogenesis, a process needed for cancer cells to meet the excess nutritional requirements of sustained cancer cell growth and subsequent cancer cell proliferation. Moreover, the Warburg effect, as a hallmark of cancer metabolism, affects glucose metabolism to further influence amino acid and lipid metabolism. Elise et al. indicated that resveratrol could reverse the Warburg effect by activating the AMPK pathway in CRC [130]. This result indicated that many signaling pathways might influence the Warburg effect by directly or indirectly activating the AMPK pathway. Therefore, whether other pathways can independently affect metabolism requires the exclusion of the effect of AMPK in future studies.

### The NF-κB signaling pathway, a central mediator between inflammation and cancer, promotes the development of CRC

The NF-κB signaling pathway has central roles in innate and adaptive cellular immunity and serves as an important link between inflammation and cell survival. In the inactivated state, a large portion of NF-κB is bound to inhibitor of NF-kB (IkB) proteins to form dimers in the cytosol; NF-κB signaling is divided into two pathways: the canonical pathway and the noncanonical pathway [29].

In the canonical NF-κB signaling pathway, NF-κB can be activated by multiple inflammatory stimuli, such as lipopolysaccharide (LPS), IL-1α/β, TNF-α and many medicines. Stimulus-specific receptors release these various inflammatory stimuli to convert into protein phosphorylation signals. The activation signal of the receptor is transmitted mainly by IκB kinase (IKK), which promotes IκB inhibitory protein degradation and phosphorylation. The activated IKK complex consists of two catalytic subunits (IKKα and IKKβ) and a regulatory component (IKKγ/NF-κB essential modulator (NEMO)). Normally, the activation of IKK is mainly mediated by IKKβ. Finally, IKK activates IκB to promote NF-κB translocation into the nucleus, where it activates target genes, such as chemokines, cytokines and antiapoptotic genes [131]. The noncanonical NF-κB pathway is primarily mediated by B cell activating factor (BAFF), CD40 ligand and lymphotoxin β/ (LTβ), which are members of the TNF family. NF-κB-inducing kinase (NIK) can promote the activation of dimerized IKKα. Subsequently, the p100 protein processing process begins. By processing p100, nuclear translocation of p52/NF-κB2 complexes induces downstream gene expression to mediate secondary lymphoid organ development and to regulate adaptive immune responses [29].

Constitutional NF-κB activation in cancer cells enhances the level of growth factor genes such as hepatocyte growth factor (HGF), BMP, and granulocyte colony-stimulating factor (G-CSF) [29]. Furthermore, NF-κB, a significant transcription factor, binds to the cyclin D1, D2, and D3 promoters to accelerate the cancer cell cycle [134]. In addition, cancer metastasis can be promoted by activating NF-κB by increasing gelatinases (MMP-2 and MMP-9) [135]. NF-κB also inhibits expression of epithelial markers E-cadherin and desmoplakin and induces expression of mesenchymal marker vimentin [136]. Tong et al. found that apigenin, a Chinese medicine monomer, inhibits the activation of NF-κB to inhibit Snail expression, which further indicated that the EMT cascade was one of the downstream targets of the NF-κB pathway [137]. On the other hand, NF-κB repression may be an effective anti-angiogenic therapy for CRC, especially in the case of NF-κB activation. Microarray and proteomic array analyses identified that several chemokines were associated with increased angiogenesis and constitutive NF-κB activation of cancer cells [138]. Inhibition of NF-κB could repress these chemokines, such as MCP-1, VEGF, IL-8 and Gro-α [138–141]. Moreover, PI3K promoted the transcription of MDM2 and degradation of p21 in an NF-κB-dependent manner, which can promote colon cancer cell migration [142].

### Ras/Raf/MEK/MAPK/ERK signaling pathway dysregulation promotes the progression of CRC

The RAS/mitogen-activated protein kinase (MAPK) signaling pathway has been widely studied in mammalian cells and plays a significant role in human cancer by promoting cell proliferation and cell cycle progression. Approximately 30% of tumors have tumorigenic mutations in the RAS gene. Activated RAS mediates RAF activation and subsequently promotes RAF phosphorylation and mitogen-activated protein kinase (MEK) activation, which promotes the phosphorylation and activation of MAPK/extracellular signal-related kinase (ERK) [143].

In CRC, gene mutations in the MAPK pathway are activating. Ganoderma lucidum polysaccharide (GLP) increases the expression of JNK via the MAPK pathway and induces the apoptosis of HCT-116 cells. GLP-induced apoptosis of human CRC cells is associated with mitochondrial and MAPK pathway activation [144]. However, in another report, activation of PIK3CA mutations decreased sensitivity to MEK inhibitors, while PTEN mutations appeared to lead to complete resistance. Furthermore, dual inhibition of the PI3K/AKT and RAF/MEK/ERK pathways also appears to completely inhibit the effect of the downstream mTOR pathway [145]. Interestingly, Wnt signaling was found to be a potential mediator of resistance to the MEK1/2 inhibitor in cancers with BRAF mutation [146], which may be induced by cell migration-inducing and hyaluronan-binding protein (CEMIP) [147]. Furthermore, another study indicated that AKT and Wnt pathways were slightly attenuated by the k-Ras inhibitors ABT263 and axitinib in k-Ras mutant CRC cells [148], which further suggested that Ras signaling could interact with AKT and Wnt pathways in CRC.

### The C-Jun N-terminal kinase (JNK) signaling pathway has a dual effect on CRC progression

The proto-oncoprotein c-Jun is a component of the AP-1 transcription factor, and its activity is enhanced in a variety of cancer types [30]. A significant mechanism by which AP-1 is activated is phosphorylation of c-Jun n-terminal kinases (JNKs) on the amino terminus of c-Jun [149]. Phosphorylation of c-Jun can form a ternary complex with TCF4 (the HMG-box transcription factor) and β-catenin. Furthermore, TCF4 could also interact on the promoter to activate c-Jun in a β-catenin-dependent manner [150]. In addition, using the Apc^Min^ mouse CRC model, inhibition of phosphorylated c-Jun [151] or c-Jun colon-specific inactivation mice attenuated the number, shape and size of cancer as well as increased their lifespan [152]. Transcriptional cooperation between TCF4 and c-Jun is activated by β-catenin and relies on TCF and AP-1 c-jun promoter elements, indicating that the interaction of phospho-c-Jun-TCF4 may promote transcription by recruiting β-catenin to the proximal transcriptional initiation site of AP-1 elements [150].

However, JNK1 is located upstream of Stat3. The JNK1-Stat3 pathway was first found to inhibit centrosomal amplification of human HCT116 cells, suggesting that it has another anticancer mechanism [150,153]. The CRC pathogenesis study conducted by Bai et al. [154] found that ZBP-89 induces apoptosis mediated by JNK activation by inhibiting JNK dephosphorylation.

Several studies have indicated that the Hippo pathway is mediated by JNK. Bleomycin can activate JNK to mediate the nuclear translocation of Yki, which enhances the expression of Upd and activates Jak-Stat signaling to accelerate cell proliferation [155,156]. Furthermore, JNK activates YAP1 in response to DNA damage [157]. JNK enhances the activation of YAP1 by inhibiting LATS by promoting the binding of Ajuba (the mammalian homologue of Jub) and LATS [158]. However, no studies have been conducted to establish a connection between the JNK signaling pathway and the Hippo pathway in the mammalian colon, which might indicate an interaction involving both the JNK pathway and the Hippo pathway in CRC tumorigenesis. In general, these results suggest that the JNK pathway may play a dual role in the carcinogenesis of CRC.

## The importance of communication among these pathways

**Figure 2:**
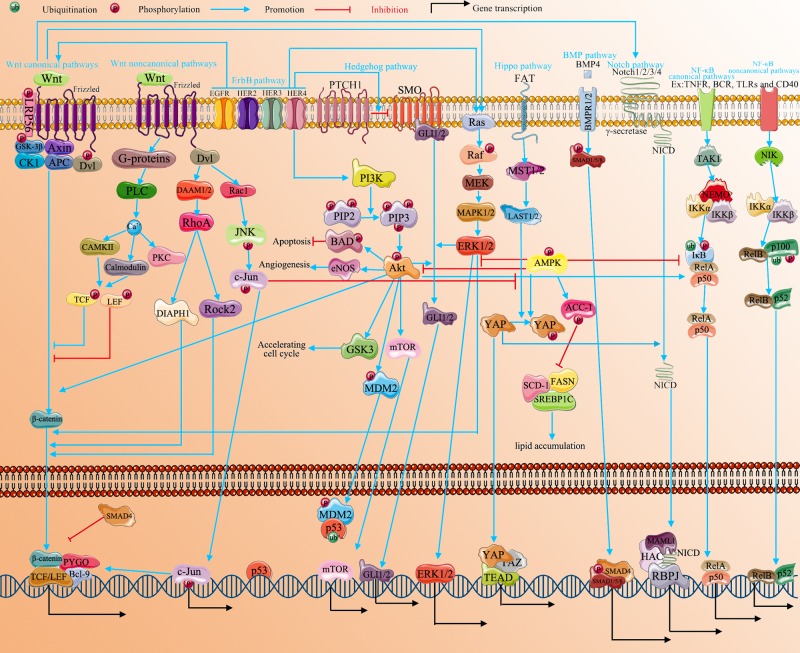
The correlation of multiple signaling pathways in CRC The development and progression of CRC is mediated by multiple signaling pathways, including Wnt, PI3K/Akt, Hedgehog, ErbB, RHOA, Notch, BMP, AMPK, Hippo, NF-κB, MAPK and JNK. These signaling pathways do not function singly but interact with each other. AMPK, BMP and Hippo mainly play anticancer roles in CRC progression. Other pathways are involved in CRC as carcinogenic factors. These pathways interact not only in the cytoplasm but also in the nucleus. These interactions regulate the transcription of downstream genes leading to cancer cell proliferation, apoptosis inhibition, migration, invasion and dedifferentiation.

The linear progression of the PI3K/Akt, Hedgehog, ErbB, RHOA, Notch, BMP, Hippo, AMPK, NF-κB, MAPK and JNK pathways is summarized in Figure 2. Nevertheless, a growing number of studies on a variety of tissues suggest that these important signaling pathways participate in cross-talk or share crucial junctions of interaction [159,160].

Wnt signaling has been reported to control the expression of YAP [27] and to regulate Gli3 from the HH pathway [102]. Furthermore, it also interacts with Notch [102], Rho [24], JNK [30] and Ras signaling [44]. PI3K/Akt [17], Wnt [19] and Ras [3] signaling can be regulated by ErbB. RAS mutations mediate the stability of the RAS protein, resulting in RAS proteins existing primarily in the GTP binding form, making them structurally active and immune to exogenous growth factors such as EGF. Thus, the mutant KRAS protein that makes up the active conformation may make tumor cells resistant to ErbB-targeted drugs, including cetuximab or panizumab [161]. Activation of the PI3K/AKT pathway can promote the NF-κB pathway to accelerate the development and progression of CRC [162]. Furthermore, k-Ras can also increase the level of NF-κB to promote carcinogenesis in CRC [163]. Activating mutations of k-Ras can increase Gli1 via the RAF/MEK/ERK and PI3K/AKT pathways [164,165]. The relationship between Wnt/β-catenin and Hedgehog/Gli was observed in CRC. These pathways are controlled by common factors, including GSK3β, p53, PTEN and KRAS. β-catenin could be increased by Gli1 via Snail and Wnt. In addition, Gli1 could also be enhanced by β-catenin though c-myc, which binds to the coding region of Gli1 mRNA to increase stability [166]. Furthermore, a previous study indicated that SMO can inhibit the activity and nuclear translocation of β-catenin, which does not rely on the function of Gli [167]. Moreover, HH can also interact with Notch by activating Hes-1 [168]. Activated AMPK can not only inhibit the activation of ErbB [23], PI3K/Akt [20,21] and Ras [23] signaling but can also promote the biological function of NF-κB [127,128]. Likewise, Hippo can be activated by AMPK [28] but inactivated by both PI3K/Akt [59] and JNK [158]. The synergistic effect on Hes-1 is switched on by Notch and HH signaling [168]. The Notch pathway can also be up-regulated by Wnt [25] and down-regulated by Hippo [121]. BMP and Wnt appear to be interconnected via the PI3k/Akt pathway [100]. Furthermore, Smad promotes EMT through Wnt, Ras, HH and Notch [169]. BMPs are also regulated by NF-κB, which indicates the importance and complexity of pathway interactions [132,133]. In addition, a recent report noted that k-Ras up-regulated ERK levels to inhibit BMP4 [132,133,170].

Therefore, the interaction among these pathways has a significant role in the formation and development of cancer. Feedback loops are also essential factors in carcinogenesis, which indicates that a slight move in one part may affect the situation as a whole.

## Conclusion

This paper reviews the pathophysiological functions, characteristics and interactions of multiple signaling pathways in CRC. A large number of pathophysiological studies have confirmed that dysregulation of signaling pathways plays a significant role in CRC, promoting cell proliferation and migration but inhibiting cell differentiation and apoptosis through multiple interactions and feedback loops [159,160].

Over the past decade, many pathways been shown to play a role in tumorigenesis, driving the development of targeted therapies. Studies have shown that the roles of these pathways are not isolated but are interconnected, with changes in one pathway leading to changes in the other [171]. By focusing on molecular cross-talk, it is possible to develop more effective therapeutic strategies.

Recent studies have suggested that the accumulation of mutations that lead to tumor heterogeneity might not act as bystanders but rather may play an active role in establishing a unique biological phenotype for each cancer patient. By understanding the interplay of pathways, we can develop effective global strategies for multiple cancers. The formation, development and progression of CRC is mediated by the dysregulation of multiple signaling pathways, including Wnt [19], PI3K/Akt [20,21], HH [22], ErbB [23], RHOA [24], Notch [25], BMP [26], Hippo [27], AMPK [28], NF-κB [29], MAPK [3] and JNK [30].

Hence, it is necessary to conduct in-depth research on the relationship between the basic etiology, inducing factors and clinical treatment of CRC to develop new therapeutic strategies with more scientific and clinical value [3]. However, if we treat CRC by targeting a single gene, we diverge from the optimal path to accurately treating CRC. Because the causes of CRC are so diverse, multidrug combinations known as ´cocktail therapy’ will promote more systematic treatment of the disease. Finally, the concept of epigenetics has been widely applied in the occurrence and development of CRC, and investigating these changes will help us explore new treatment options and improve the early diagnosis and treatment of CRC [172].

## Abbreviations

AGE: advanced glycation end product
CRC: colorectal cancer
EMT: epithelial–mesenchymal transition
ROS: reactive oxygen species
UPR: unfolded protein response
